# Precision and prediction matter: investigating hearing recovery measurements and prognosis in sudden sensorineural hearing loss

**DOI:** 10.1007/s00405-025-09675-4

**Published:** 2025-10-06

**Authors:** Emilia Nordlie, Johanna Elander, Maria Värendh, Karin Stenfeldt, Fredrik Tjernström, Marie Gisselsson-Solén, Julia Sjögren, Måns Magnusson, Johannes K. Ehinger

**Affiliations:** 1 Otorhinolaryngology, Department of Clinical Sciences, Lund University, Lund, Sweden; 2 School of Medical Sciences, Faculty of Medicine and Health, Department of Otorhinolaryngology, Örebro University Hospital, Örebro University, Örebro, Sweden; 3 Logopedics, Phoniatrics and Audiology, Department of Clinical Sciences, Lund University, Lund, Sweden; 4 Mitochondrial Medicine, Department of Clinical Sciences, Lund University, Lund, Sweden; 5 Department of Otorhinolaryngology, Head and Neck Surgery, Skåne University Hospital, Lund, Sweden

**Keywords:** Sudden sensorineural hearing loss, Prognosis, Corticosteroids, Audiometry, Treatment

## Abstract

**Purpose:**

Sudden sensorineural hearing loss (SSNHL) is defined as 30 decibels (dB) hearing loss in 3 consecutive frequencies occurring within 72 h. Pure-tone average of four frequencies (PTA4) is commonly used to evaluate hearing levels but may not accurately reflect the recovery. We aimed to identify prognostic factors for recovery and to evaluate how recovery should be assessed, by comparing PTA4 with an individual pure-tone average (iPTA), including solely the hearing thresholds for the affected frequencies.

**Methods:**

Demographic, clinical, and audiologic factors were analyzed using multivariable linear and logistic regression models. A Bland-Altman plot was used to compare recovery measurements based on iPTA and PTA4.

**Results:**

In this cohort, the mean age was 57 years (range 19–91 years). Dizziness was a prominent negative predictive factor (logistic regression: iPTA OR 0.09 95% CI 0.02–0.38, for full recovery; linear regression: iPTA 14.4 dB poorer recovery). Tinnitus correlated with, on average, 4.9 dB poorer recovery (*P*=0.043). Each day of delayed assessment was linked to a 0.84 dB reduction in recovery (*P* <0.001; OR 0.92, 95% CI 0.87–0.98). Comparing PTA4 with iPTA, the Bland-Altman plot showed −2.4 dB mean difference with wide limits of agreement, ranging from approximately −17 to 13 dB. In cases of frequency range-specific hearing loss, hearing recovered 8.7 dB more by using iPTA than PTA4 (*P*=0.003).

**Conclusion:**

Dizziness, tinnitus, and increasing disease duration until assessment are negative prognostic factors. Compared to PTA4, iPTA better reflects actual hearing recovery, particularly in frequency range-specific hearing loss.

**Supplementary Information:**

The online version contains supplementary material available at 10.1007/s00405-025-09675-4.

## Introduction

Sudden sensorineural hearing loss (SSNHL) is defined as at least 30 decibels (dB) hearing loss (HL) across 3 or more consecutive frequencies on a pure-tone audiogram, developing within 72 h [1]. Approximately 98% of cases are unilateral, and the condition is frequently associated with dizziness and tinnitus [1]. The incidence is estimated to 10–30 per 100 000, with an incidence peak at age 50–60 years, but individuals of all ages may be affected [2–5]. Due to the lack of large epidemiological studies and frequent spontaneous recovery, the true incidence is unknown. Numerous etiologies have been proposed, including vascular impairment, infectious conditions, autoimmune disease, traumatic injury and neoplastic disease [1, 6, 7]. For most affected individuals, the underlying pathophysiology is enigmatic [7]. Either the cochlea or retrocochlear structures may be affected. SSNHL of retrocochlear origin is typically due to a benign cerebellopontine tumor, usually a vestibular schwannoma, but other conditions, such as viral infection, Lyme disease, multiple sclerosis, or stroke may also affect the vestibulocochlear nerve or central auditory nervous pathways [7–10].

Different treatment modalities have been tested based on various hypothesized pathophysiologies. Corticosteroids are thought to suppress immunologically mediated conditions causing SSNHL [11], and standard treatment is high-dose oral corticosteroids for 10–14 days [1]. Intratympanic corticosteroid injection may be considered, but is primarily used as salvage treatment, or if oral corticosteroids are contraindicated [1]. Hyperbaric oxygen therapy has also been suggested as treatment [1]. However, all tested treatment modalities lack evidence of efficacy [11–14]. Spontaneous recovery occurs in up to 65% of the cases, which leads to difficulties in clinical trial design [1, 15]. Furthermore, diverse criteria for hearing recovery, inconsistent number of evaluated frequencies, and variously calculated pure-tone averages (PTA) limit the possibility of comparing studies [15].

Studies suggest that early start of oral corticosteroid treatment, and low- or mid-frequency HL are prognostically favorable, while advanced age, dizziness, family history of HL, and severe initial HL are negative prognostic factors [6, 16–19]. There is still ambiguity regarding the prognostic value of parameters such as comorbidities and tinnitus [6, 16, 19, 20].

The aim of this study was to retrospectively evaluate prognostic factors for recovery in a cohort of SSNHL patients seen over 5 years at a tertiary referral center for otorhinolaryngology. As a second aim, we set out *to specifically assess the frequencies affected by SSNHL*, in addition to the commonly used pure-tone average of the frequencies 500, 1000, 2000, and 4000 Hz (Hz) (PTA4) [1, 21], to possibly evaluate hearing recovery with higher precision. Compared to an individualized recovery measurement, we hypothesized PTA4 to be imprecise, since some frequencies affected by the disease may not be evaluated. To our knowledge, hearing recovery in SSNHL has not previously been evaluated solely based on the consecutive affected frequencies.

## Methods

### Patients

Following approval by the Swedish Ethical Review Authority (DNR 2021 − 00990) a retrospective audit of medical records was performed for all patients diagnosed with ICD-10 code H91.2 (sudden idiopathic hearing loss) at Skåne University Hospital, Lund, Sweden, between January 2016 and December 2020, in total 562 patients.

The retrieved data included: demographic; audiometric; treatment given; comorbidities; subjective tinnitus and/or dizziness; and time aspects. Standard treatment was 60 mg oral prednisolone daily for five days, followed by a five-day taper-off period. In some cases, the corticosteroid dose was increased/prolonged or reduced. In the analyses, any corticosteroid treatment was compared to no treatment. Disease duration until assessment was defined as the number of days from HL onset to treatment initiation, or to the diagnostic audiogram if no treatment was given. Non-idiopathic cases were included in the study to reflect typical clinical settings and to reduce the risk of look-ahead bias. Subjectively reported dizziness was defined as a tangible sensation of dizziness within a day from HL onset. Age was categorized into young adults (18–39 years), middle-aged adults (40–59 years), older adults (60–69 years), and elderly (≥ 70 years).

### Inclusion and exclusion criteria

The main diagnostic criterion was ≥ 30 dB HL in ≥ 3 consecutive frequencies, occurring within 72 h. If the HL had an undeterminable onset or appeared more than 30 days before the initial assessment, the individuals were excluded. Air- and bone-conduction in PTA4 was manually compared on the diagnostic audiogram, and all patients with primarily conductive HL were excluded, as were individuals with a pre-known condition causing HL, or if treatment was initiated > 3 days before the initial audiogram. All patients lost to audiometric follow-up or without an initial audiogram were excluded. Individuals aged < 18 years were excluded.

### Audiometry

Pure-tone hearing thresholds at frequencies 125, 250, 500, 1000, 1500, 2000, 3000, 4000, 6000, and 8000 Hz were obtained from medical records. Hearing thresholds at 500, 1000, 2000, 4000, and 6000 Hz were available for all patients, while data for other frequencies were missing in some cases. The hearing thresholds were compared to the contralateral ear. In this study, all consecutive affected frequencies, that fulfilled the main criterion (≥ 30 dB HL in ≥ 3 consecutive frequencies), were calculated to an individual pure-tone average (iPTA). The initial degree of HL was categorized, in accordance with the World Health Organization’s classification, to no/mild (≤ 40 dB PTA4), moderate (41–60 dB PTA4), severe (61–80 dB PTA4), and profound (> 80 dB PTA4) HL [22]. Low-frequency HL was defined as ≥ 30 dB HL at frequencies 125, 250, and 500 Hz, while high-frequency HL included the frequencies ≥ 3000, 4000, 6000, and 8000 Hz. Frequency range-specific HL was defined as ≥ 30 dB HL at three or four consecutive frequencies, without ≥ 30 dB HL at any other frequency. Hearing outcome was evaluated in regard to full and partial recovery, respectively, following the recommendations in *Clinical Practice Guideline: Sudden Hearing Loss* [1]; full recovery was defined as a return to ≤ 10 dB iPTA compared to the contralateral ear for the same frequencies; all other cases were considered as incomplete recovery. Partial recovery was defined as recovery > 10dB but not fulfilling the criterion of full recovery. No recovery was defined as ≤ 10dB. In addition to recovery in iPTA, similar categorizations were performed for PTA4 values.

### Statistical analyses

Statistical analyses were performed using SPSS Statistics 29.0 (IBM Corp, Armonk, NY, USA). Following visualization in histograms and evaluating skewness, data was assumed to be normally distributed. Prognostic factors were analyzed in two separate multivariable linear regression models, using absolute iPTA and PTA4 recovery values in dB, respectively, as dependent variables. The categorical recovery outcomes were analyzed using multivariable logistic regressions, using the recovery definitions, separately, as the dependent variable; full recovery was compared to incomplete recovery while partial recovery was compared to no recovery. The independent variables in the logistic regression models were selected from the multivariable linear regression model; the most indicative factors, with assumed potential to affect hearing outcome, were included. Subanalyses with simple linear regressions were performed to evaluate relationship between hearing recovery and disease duration until assessment for the corticosteroid treated and untreated group respectively; adjusted linear regressions were performed based on which variables were unevenly proportioned between the treated and untreated groups. Using unpaired t-test, recovery measured in iPTA and PTA4 respectively was compared in frequency range-specific HL. A Bland-Altman plot was used to assess agreement between PTA4 and iPTA [23]; with iPTA selected as the reference method based on the hypothesis that it more accurately estimates hearing recovery [24]. The difference between PTA4 and iPTA in each individual was plotted against iPTA recovery to compare the measurements; simple linear regression was performed to analyze correlation. One-way ANOVA was performed for mean hearing recovery in dB per affected frequency. A two-sided p-value < 0.05 was considered statistically significant. Figures were made using Graph Pad Prism 10.1 (GraphPad Software, Boston, MA, USA).

## Results

**Fig. 1:**
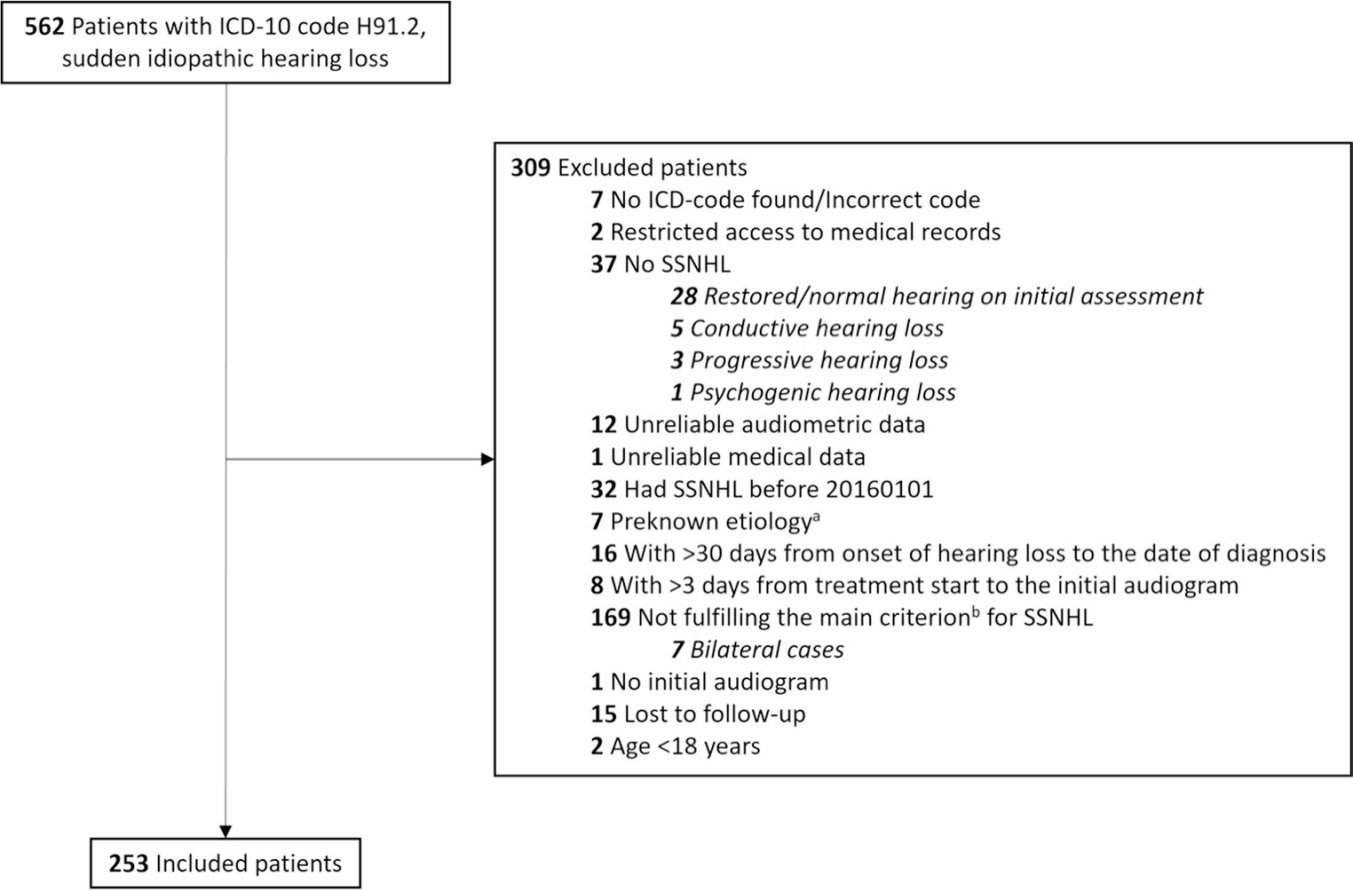
Study flowchart. Abbreviations: SSNHL: Sudden sensorineural hearing loss; ICD: International Classification of Diseases. ^a^The preknown etiologies were acoustic-, physical- or barotrauma, known Mb Ménière, and labyrinthitis as a complication of acute otitis media. ^b^The criterion for SSNHL is at least 30 decibels hearing loss in at least 3 consecutive frequencies

In total, 253 individuals fulfilled the inclusion criteria while not meeting the exclusion criteria (Fig. 1). Clinical characteristics of the study patients are presented in Table 1. The mean age was 57 years (range 19–91 years). Comorbidities present in at least 10% of the cohort were hypertension (36%) and diabetes mellitus (13%). An etiology for SSNHL was subsequently identified in 17 patients (7%): four cases of Mb Ménière; Lyme disease and varicella zoster in three cases each; vestibular schwannoma in two cases; and autoimmune disease, herpes simplex, superior canal dehiscence syndrome, infectious labyrinthitis, and vasculitis in one case respectively. Tinnitus was present in 50% of the cases, while dizziness was an associated symptom in 23%. Two hundred seven patients (82%) received corticosteroids, while 46 (18%) received no treatment. The majority of patients (74%) were given first-line, standard treatment.

### Hearing recovery in iPTA

**Fig. 2:**
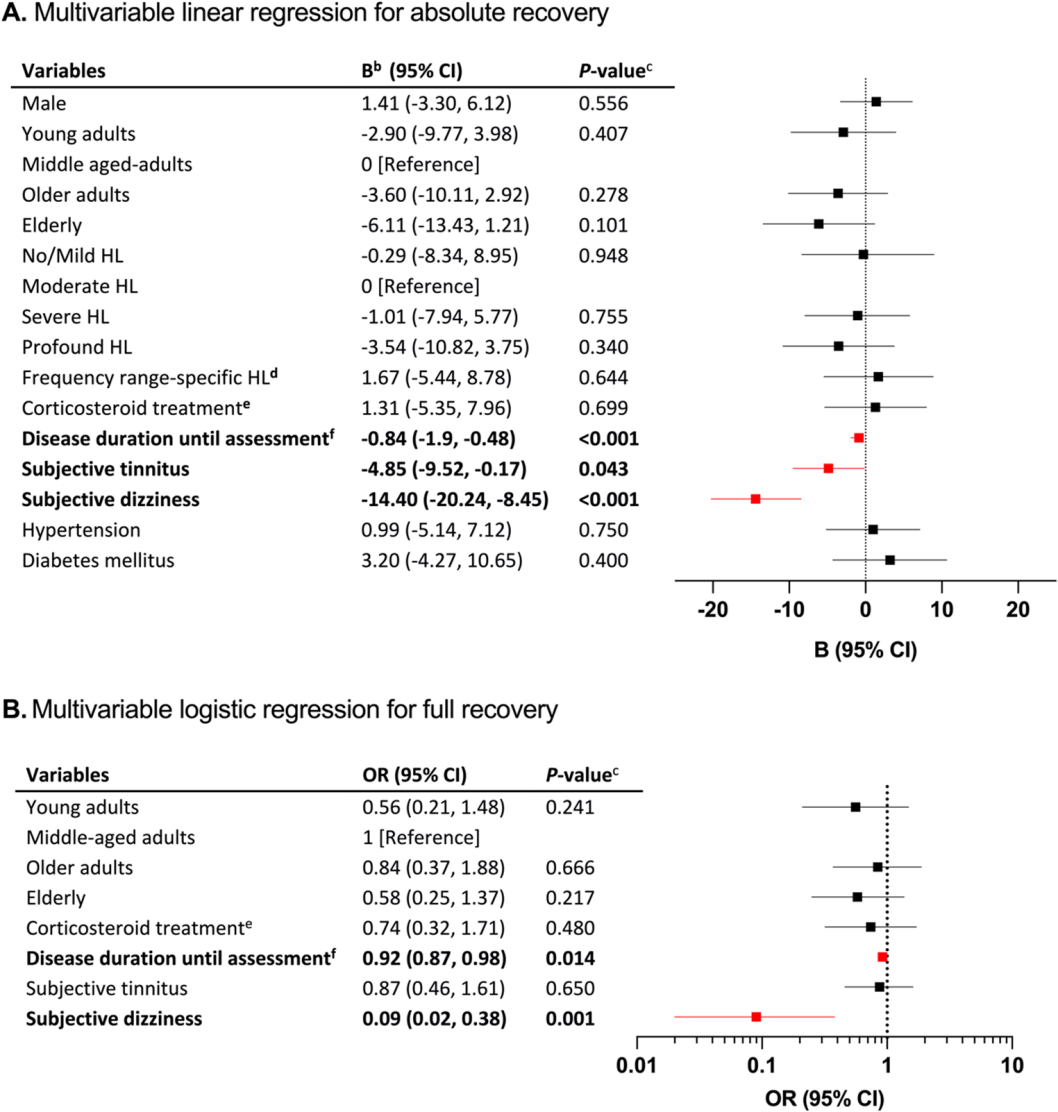
Forest plots of prognostic factors for hearing recovery^a^. Abbreviations: CI: Confidence interval; HL: hearing loss; h: hours; dB: decibels; OR: odds ratio. OR is displayed as logarithmic data for the purpose of data visualization. Age categories: Young adults: 18–39 years; Middle-aged adults: 40–59 years; Older adults: 60–69 years; Elderly: ≥70 years. Degree of HL: No/mild HL: ≤40 dB pure tone average of the frequencies 500, 1000, 2000, and 4000 Hz (PTA4); Moderate HL: 41–60 dB PTA4; Severe HL: 61–80 dB PTA4; Profound HL: >80 dB. ^a^Hearing recovery in A is defined as the absolute improvement in individual pure-tone average (iPTA), including solely affected frequencies, in B is defined as ≤ 10 decibels pure tone average of affected frequencies compared to the contralateral ear. ^b^B indicates the change in the outcome variable per 1 unit of change in the covariate. For categorical variables, B represents the effects of moving from the reference category. ^c^*P-*value < 0.05 is considered statistically significant.^d^Frequency range-specific HL: ≥30 dB hearing loss in solely 3 or 4 consecutive frequencies. ^e^No treatment as reference ^f^Disease duration until assessment was defined as days from onset of HL to treatment start or, if no treatment was given, days to diagnostic audiogram.

**Fig. 3:**
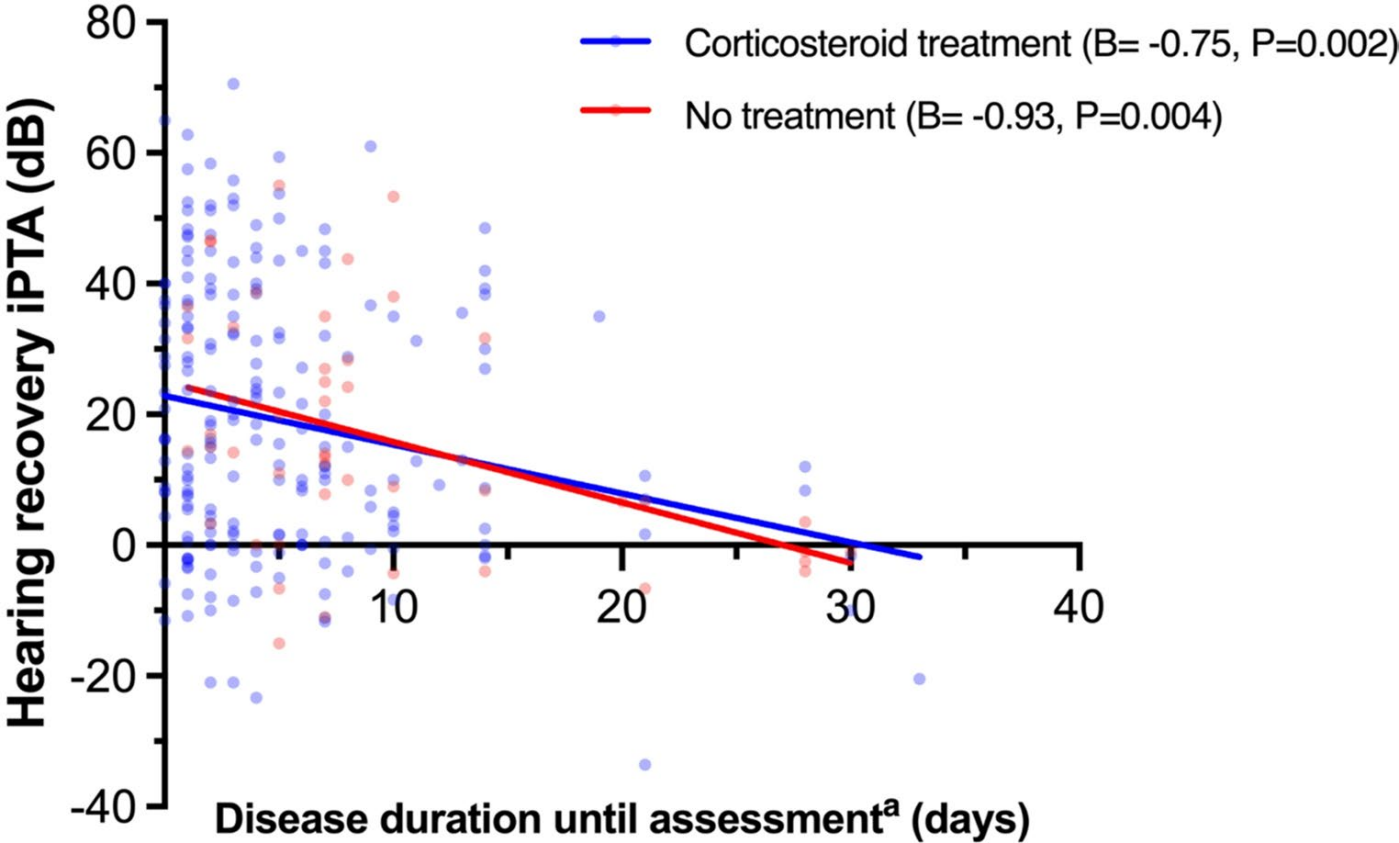
Linear regressions for evaluation of relationship between hearing recovery and disease duration until assessment for corticosteroid treated and untreated. The blue line indicates simple linear regression with hearing recovery in iPTA as dependent variable, analyzing days to treatment start (B*=* −0.75, CI 95% −1.211, −0.283). The red line represents simple linear regression of time to diagnostic audiogram, with no treatment given (B*=* −0.93, CI 95% −1.531, −0.322). ^a^Disease duration until assessment was defined as days from onset of HL to treatment start (blue), or, if no treatment was given, days to diagnostic audiogram (red).

**Fig. 4:**
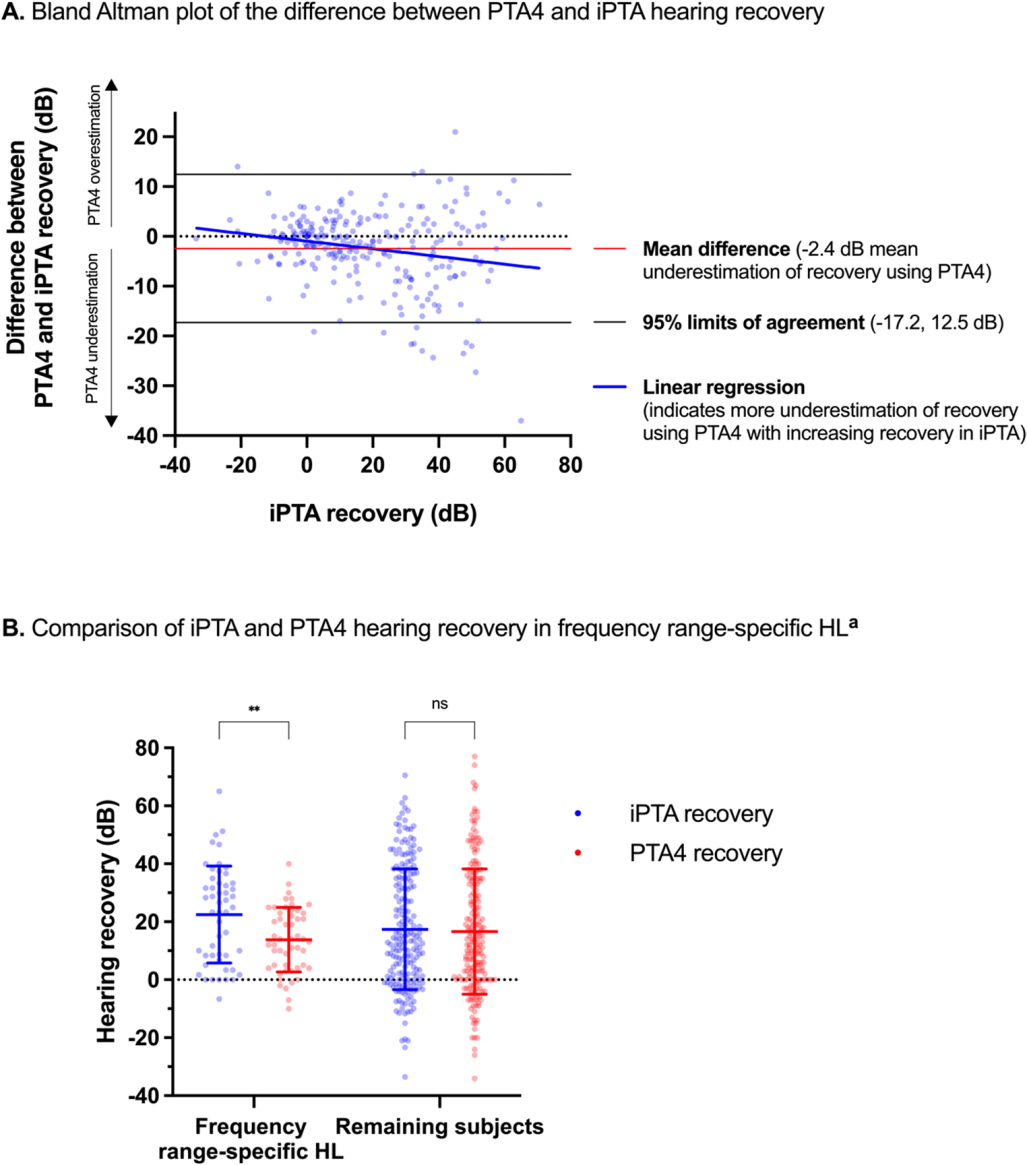
Comparisons of iPTA and PTA4 hearing recovery. Abbreviations: iPTA: individual pure-tone average of solely affected frequencies; PTA4: pure-tone average of four frequencies, 500, 1000, 2000, and 4000 Hz; dB: decibels; HL: hearing loss. A, Method comparison using Bland-Altman plot, comparing the hearing recovery measurements iPTA and PTA4; iPTA is selected as reference method. The reference lines show: mean difference between PTA4 and iPTA recovery (red line); 95% limits of agreement, ± 1.96 SD (black lines); simple linear regression of iPTA recovery, with difference between recovery in PTA4 and iPTA as dependent variable (blue line, B= −0.08, 95% CI −0.1, −0.03, *P* = 0.001). B, Multiple unpaired t-tests. Significant mean difference of 8.7 dB between hearing recovery using iPTA and PTA4 in frequency range-specific HL (*P* = 0.003). Error bars represent standard deviation. ^a^Frequency range-specific HL is defined as ≥ 30 dB hearing loss in solely 3 or 4 consecutive frequencies. ** = *P*-value < 0.01

Fifty-eight patients (23%) met the criterion for full recovery according to iPTA; among these, only two patients (3%) experienced dizziness. Subjective dizziness correlated with, on average, 14.4 dB poorer recovery and an odds ratio (OR) of 0.09 (95% CI 0.02–0.38) for full recovery (Fig. 2, *P* ≤ 0.001). Subjective tinnitus was associated with poorer absolute recovery (−4.9 dB, *P* = 0.043). Increasing disease duration until assessment was a negative predictive factor (Fig. 2, −0.84 dB, *P* < 0.001; OR 0.92 95% CI 0.87–0.98, respectively). In the unadjusted model, disease duration until assessment in the corticosteroid treatment and no treatment groups, respectively, showed similar effect sizes for hearing recovery with an average of 0.75 dB and 0.93 dB poorer recovery per day (Fig. 3, *P* = 0.001, *P* = 0.004, respectively). Based on imbalanced variables between the treated and untreated (Table S1), multivariable linear regressions were performed to adjust for age groups, degree of HL, hypertension, diabetes mellitus, tinnitus, and vertigo; Following adjustment, disease duration until assessment was associated with slightly more pronounced negative correlations (Corticosteroids: −0.82 dB, 95% CI −1.25, −0.34, *P* < 0.001, No treatment: −0.99 dB, 95% CI −1.62, −0.36, *P* = 0.003). Ninety-five patients (38%) recovered partially using iPTA. Subjective dizziness and increasing disease duration until assessment was associated with poor prognosis for partial recovery (OR 0.36, 95% CI 0.18–0.72; OR 0.91, 95% CI 0.87–0.96, respectively, Fig. S2A). The mean difference in hearing recovery between PTA4 and iPTA was − 2.4 dB (95% Limits of Agreement − 17.2 dB, 12.5 dB, Fig. 4A). Linear regression of the Bland Altman plot showed a negative correlation between iPTA recovery and the difference between PTA4 and iPTA (B=−0.08, *P* = 0.001). One-way ANOVA of affected frequencies showed a significant difference in mean hearing recovery between 500 and 6000 Hz, and 250, 500, 1000 Hz, respectively, compared with 8000 Hz (Fig. S5, *P* < 0.05)

**Table 1:** Clinical characteristics of patients

Characteristic	All patients		Full recovery^a^		Incomplete recovery	
	Mean (SD)	No. (%)	Mean (SD)	No. (%)	Mean (SD)	No. (%)
Age (years)	57 (±17)	253	56 (±16)	58	58 (±18)	195
Young adults (18–39)		51 (20)		8 (14)		43 (22)
Middle aged adults (40–59)		66 (26)		18 (31)		48 (25)
Older adults (60–69)		67 (27)		18 (31)		49 (25)
Elderly (≥ 70)		69 (27)		14 (24)		55 (28)
Sex						
Female		115 (46)		19 (33)		96 (49)
Male		138 (55)		39 (67)		99 (51)
Affected ear						
Right		109 (43)		26 (45)		83 (43)
Left		144 (57)		32 (55)		112 (57)
Comorbidity						
Hypertension		91 (36)		19 (33)		72 (37)
Diabetes mellitus		34 (13)		7 (12)		27 (14)
Associated symptoms						
Subjective tinnitus		127 (50)		27 (47)		100 (51)
Subjective dizziness		57 (23)		2 (3)		55 (28)
Initial iPTA (dB)	71 (±16)		61 (±13)		75 (±15)	
Initial PTA4 (dB)	68 (±23)		53 (±22)		75 (±21)	
Degree of HL (PTA4, dB)						
No/Mild HL (≤ 40)		38 (15)		13 (22)		25 (13)
Moderate HL (41–60)		51 (20)		19 (33)		32 (16)
Severe HL (61–80)		80 (32)		19 (33)		61 (31)
Profound HL (≥ 81)		84 (33)		7 (12)		77 (39)
Frequency range-specific HL^b^		51 (20)		20 (34)		31 (16)
Disease duration until assessment^c^	6 (7)		4 (4)		7 (7)	
Days to treatment	5 (6)		4 (4)		6 (6)	
Days to diagnostic audiogram, if no treatment was given	10 (8)		6 (4)		11 (9)	
First-line treatment						
Standard treatment^d^		188 (74)		45 (78)		143 (73)
Increased oral corticosteroids		3 (1)		0 (0)		3 (2)
Reduced oral corticosteroids		9 (4)		1 (2)		8 (4)
Intratympanic corticosteroids		7 (3)		1 (2)		6 (3)
No treatment given		46 (18)		11 (19)		35 (18)

*SD* Standard deviation; *No* number of patients; *HL* hearing loss; *PTA*4 : 4 frequencies pure-tone average at 500, 1000, 2000, and 4000 Hz hearing thresholds; iPTA: individual pure-tone average, average of affected frequencies; *dB* decibels; *h* hours

All the % is of column total

^a^Full hearing recovery is defined as ≤ 10 decibels individual pure-tone average of affected frequencies compared to the contralateral ear

^b^Frequency range-specific HL is ≥ 30dB hearing loss in solely 3 or 4 consecutive frequencies

^c^Disease duration until assessment was defined as days from onset of HL to treatment start or, if no treatment was given, days to diagnostic audiogram

^d^Treatment with 60 mg oral corticosteroids for 5 days followed by 5 days taper-off period

### Hearing recovery in PTA4

When using PTA4, 75 patients (30%) recovered fully. In three of these cases, the criterion for full recovery according to PTA4 had already been fulfilled at the time of diagnosis. Subjective dizziness was associated with poor prognosis (13.6 dB poorer average recovery and OR 0.11, 95% CI 0.04–0.34, of full recovery, Fig. S1, *P* < 0.001). Disease duration until assessment correlated with poorer outcome in PTA4 (−0.78 dB poorer recovery per day elapsed; OR 0.92 95% CI 0.87, 0.97, *P* < 0.001 respectively). In contrast with the regression analyses using iPTA, being elderly was associated with 7.8 dB poorer recovery compared to the middle-aged group (*P* = 0.041, Fig. S1A), and with an OR of 0.36 (95% CI 0.16–0.82, Fig. S1B) for full recovery. Sixty-nine patients (27%) recovered partially using PTA4. Subjective dizziness, tinnitus, and disease duration until assessment correlated with poorer recovery (OR 0.35, CI 95% 0.16–0.74; OR 0.47 CI 95% 0.24–0.91; OR 0.95, CI 95% 0.90–0.99, Fig. S2B).

### Hearing recovery in frequency range-specific hearing loss

Fifty-one patients (20%) had frequency range-specific HL. A mean difference of 8.7 dB between hearing recovery using iPTA and PTA4 in frequency range-specific HL was found (Fig. 4B, *P* = 0.003). No significant difference was found in low- or high-frequency HL when analyzed separately (Fig. S6).

### Sensitivity analyses

Thirty-eight patients initiated corticosteroid treatment prior to the initial audiogram, and a sensitivity analysis was performed to control for this by repeating the linear regression with iPTA recovery as dependent variable while excluding these individuals. Results were similar to the primary analysis (Fig. S3A), except that tinnitus became insignificant. In 19 cases, the treatment deviated from clinical standard; excluding these cases did not affect the main results (Fig. S3B). A sensitivity analysis excluding the 17 cases with an identified etiology after the diagnosis of SSNHL yielded results similar to the main analysis (Fig. S4).

## Discussion

### PTA4 or an individual PTA specifically including the affected frequencies?

In this study, an individual PTA was calculated using the hearing thresholds for the affected frequencies. To the best of our knowledge, this approach has previously never been used to evaluate recovery and prognosis. Classically, a PTA with a predefined set of frequencies is used and consequently, some affected frequencies may not be evaluated. The inclusion criteria for SSNHL are not consistently specified across studies; in particular, information on what frequencies were required for inclusion is often missing. Moreover, the definition of hearing recovery varies widely in the literature [21], and a meta-analysis identified this heterogeneity in PTA definitions as problematic when comparing studies [25]. Here, we sought to define and evaluate recovery in a way that corresponds to the diagnostic criterion of SSNHL and to capture recovery more accurately. Illustrating this discrepancy and highlighting the need for a more personalized measure of outcome, three included SSNHL cases already fulfilled the criterion for “full recovery” as measured by PTA4 at the time of diagnosis, however, not by iPTA. Further demonstrating this, a greater recovery as measured by iPTA was associated with an underestimation of recovery by PTA4 (Linear regression slope, B= −0.08, Fig. 4 A). Hence, PTA4 may incorrectly *underestimate* actual recovery in dB while *overestimating* full recovery. Moreover, the heteroscedasticity in the Bland-Altman plot indicates an increasing variability between PTA4 and iPTA recovery with increasing actual recovery in iPTA, further showing the bluntness of PTA4 (Fig. 4 A). The wide agreement intervals, ranging from approximately − 17 dB to 13 dB, illustrate the large variation of differences, and hence, considerably low agreement between recovery in PTA4 and iPTA. When comparing hearing recovery between the affected frequencies, we found that low-mid frequencies (250–1000 Hz) recovered better compared to high frequencies (6000–8000 Hz), which is in line with a previous study [26]. In our study, these high frequencies were affected in more than 50% of the cases, and should therefore not be omitted when assessing recovery.

### PTA4 underestimates hearing recovery in frequency range-specific hearing loss

Fifty-one patients (20%) had frequency range-specific HL in this study; In these patients, there was a difference in recovery of 8.7 dB between iPTA and PTA4, with PTA4 underestimating recovery. We believe a frequency specific reporting standard, such as iPTA, offers more precise information and reduces bias when analyzing hearing recovery.

### To treat or not to treat with corticosteroids?

While generally accepted as the standard of care, the effect of corticosteroid treatment is questionable [13, 27]. Four separate meta-analyses have failed to show a benefit of corticosteroids compared to placebo or other treatment modalities [11, 12, 25, 28] While this study was not designed to evaluate efficacy of treatment, we noted that corticosteroid treatment did not correlate with better recovery compared to no treatment. Moreover, the percentages of patients with full recovery, both by iPTA and by PTA4, were similar between the groups (Table S1). However, the non-random assignment of patients to corticosteroid treatment or no treatment constitutes substantial selection bias; untreated were, on average, slightly older, assessed later, had diabetes and hypertension more frequently, and were not as dizzy (Table S1). In this study, we found that the longer the disease duration was until assessment, the worse the hearing recovery. Furthermore, the separate linear regressions for corticosteroid treated and untreated had comparable coefficients (Fig. 3), suggesting that regardless of whether corticosteroid treatment was given or not, the earlier in the course of the disease the higher the probability of recovering. Thus, early recovery is not necessarily an effect of the corticosteroids per se, but may reflect a higher probability of spontaneous recovery in the early course of the disease [6, 29]. Furthermore, due to the different proposed etiologies and possible pathogeneses, it is reasonable that one specific treatment modality does not affect all cases.

### Dizziness

Subjective dizziness was a prominent predictive factor, which on average resulted in 14.4 dB poorer recovery, and 11 times the odds of not recovering fully, using iPTA. Results using PTA4 were similar. This is in agreement with a meta-analysis, in which dizziness was associated with poorer recovery [30]. The extent of vestibular dysfunction seems to correlate with the severity of cochlear dysfunction [20, 31], and dizziness has been associated with profound HL [32–34]. Concomitant dizziness in SSNHL patients may reflect a more extensive inner ear impairment, also affecting the vestibular organ due to the anatomical proximity and continuous membrane structures [35]. Furthermore, the cochlea and vestibule are functionally associated, both using similar sensory hair cells, which, hypothetically, make them vulnerable to the same pathological processes [36]. Our results cannot be used to determine the pathomechanism, but suggest that SSNHL with concurrent dizziness is a disease entity different from pure SSNHL, as the two conditions differ not only in symptomatology, but also in expected outcome.

### Tinnitus and age

Tinnitus is reported in 41–93% of SSNHL-cases [6, 17, 37–39], but its association to degree of injury to the inner ear is controversial, and tinnitus has been reported as an insignificant, negative and positive prognostic factor [6, 17, 40–42]. Here, 50% of the patients reported tinnitus and it correlated with poorer recovery. Since the prevalence of tinnitus in our study is lower compared to most of the literature, it is reasonable to believe that in cases tinnitus was reported, it generally was perceived as more severe or disturbing. Hypothetically, certain tinnitus characteristics may have higher proportion in our cohort, hence, be associated with poorer outcome.

Advanced age is commonly reported as a negative prognostic factor [16, 40, 43]. Most studies define elderly as over 60 years, but despite us defining elderly as ≥ 70 years, advanced age could not be unambiguously associated with a poor prognosis, as our results differed depending on whether we used iPTA or PTA4 [6].

### Limitations

Inherent limitations to retrospective study designs are the lack of data in medical records and the risk of recall bias. Dizziness was subjectively reported and vestibular function was not evaluated. The non-random assignment of patients to treatment or no treatment, and occasional loss of follow-up constitute potential selection bias. Furthermore, treatment was occasionally administered before the initial audiogram. When analyzing audiologic outcomes, statistically significant, yet minor, recovery in PTA may not necessarily be clinically relevant. Speech audiometry was not available for this cohort.

## Conclusions

Subjective dizziness, tinnitus and, increasing disease duration until assessment are negative prognostic factors for hearing recovery following SSNHL. Early recovery may be the natural history for an abundance of SSNHL-patients. iPTA better reflects recovery after SSNHL in the individual patient compared to PTA4.

## Supplementary Information

Below is the link to the electronic supplementary material.

Supplementary Material 1

## Data Availability

Data is not shared openly to protect study participants’ privacy. Additional data is available on reasonable request.
