# Supplementary material for: Precision and prediction matter: investigating hearing recovery measurements and prognosis in sudden sensorineural hearing loss
Source: Eur Arch Otorhinolaryngol. 2025 Oct 6;283(1):205–14. doi: 10.1007/s00405-025-09675-4 (PMC12904936; doi:10.1007/s00405-025-09675-4)
Supplement: Supplementary file 1 — Supplementary Material 1 [file 405_2025_9675_MOESM1_ESM.docx]

**Fig. S1 Forest plots of prognostic factors for hearing recovery in PTA4^a^**

**A.** Multivariable linear regression for absolute recovery in dB

**B.** Multivariable logistic regression for full recovery

Abbreviations: CI: Confidence interval; HL: hearing loss; h: hours; dB: decibels; OR: odds ratio

ORs are displayed as logarithmic data for the purpose of data visualization

Age categories: Young adults: 18-39 years; Middle-aged adults: 40-59 years; Older adults: 60-69 years; Elderly: ≥70 years

Degree of HL: No/mild HL: ≤40 dB pure tone average of the frequencies 500, 1000, 2000, and 4000 Hz (PTA4); Moderate HL: 41-60 dB PTA4; Severe HL: 61-80 dB PTA4; Profound HL: >80 dB

^a^ Hearing recovery in A is defined as the absolute improvement in PTA4. In B, full recovery is defined as ≤10 decibels PTA4 compared to the contralateral ear

^b^ B indicates the change in the outcome variable per 1 unit of change in the covariate. For categorical variables, B represents the effects of moving from the reference category

^c^ P-value <0.05 is considered statistically significant

^d^ Frequency range-specific HL: ≥30dB hearing loss in solely 3 or 4 consecutive frequencies

^e^ No treatment as reference

^f^ Disease duration until assessment was defined as days from onset of HL to treatment start or, if no treatment was given, days to diagnostic audiogram

**Fig. S2 Prognostic factors for partial hearing recovery**

**A.** Multivariable logistic regression, using iPTA^a^

**B.** Multivariable logistic regression, using PTA4^c^

Abbreviations: CI: Confidence interval; h: hours; PTA4: 4 frequencies pure-tone average at 500, 1000, 2000, and 4000 hertz hearing thresholds; iPTA: individual pure-tone average, average of affected frequencies

Partial recovery is compared to no recovery (≤10 dB hearing recovery)

Number of included patients in A: 195

Number of included patients in B: 178

Age categories: Young adults: 18-39 years; Middle-aged adults: 40-59 years; Older adults: 60-69 years; Elderly: ≥70 years

^a^ Partial hearing recovery is defined as hearing recovery >10 decibels iPTA but not fulfilling criterion of full recovery (≤10 decibels iPTA compared to the contralateral ear)

^b^ P-value <0.05 is considered statistically significant.

^c^ Partial hearing recovery is defined as hearing recovery >10 decibels PTA4 but not fulfilling criterion of full recovery (≤10 decibels PTA4 compared to the contralateral ear)

^d^ No treatment as reference

^e^ Disease duration until assessment was defined as days from onset of HL to treatment start or, if no treatment was given, days to diagnostic audiogram ^d^ Frequency range-specific HL: ≥30dB hearing loss in solely 3 or 4 consecutive frequencies

**Fig. S3 Sensitivity analyses of corticosteroid treatment for recovery^a^**

**A.**  Excluding all cases with treatment prior to initial audiogram

**B.**  Excluding all cases with corticosteroid treatment not according to clinical guidelines

Abbreviations: CI: Confidence interval; HL: hearing loss; h: hours; dB: decibels

Linear regressions for recovery in absolute iPTA dB (PTA of affected frequencies).

Number of included patients in A: 215

Number of included patients in B: 234

Age categories: Young adults: 18-39 years; Middle-aged adults: 40-59 years; Older adults: 60-69 years; Elderly: ≥70 years

Degree of HL: No/mild HL: ≤40 dB pure tone average of the frequencies 500, 1000, 2000, and 4000 Hz (PTA4); Moderate HL: 41-60 dB PTA4; Severe HL: 61-80 dB PTA4; Profound HL: >80 dB

Frequency range-specific HL: ≥30dB hearing loss in solely 3- or 4 consecutive frequencies

^a^ Hearing recovery is defined as the absolute improvement in individual pure-tone average (iPTA), including solely affected frequencies.

^b^ B indicates the change in the outcome variable per 1 unit of change in the covariate. For categorical variables, B represents the effects of moving from the reference category

^c^ P-value <0.05 is considered statistically significant

^d^ Frequency range-specific HL: ≥30dB hearing loss in solely 3 or 4 consecutive frequencies

^e^ No treatment as reference

^f^ Disease duration until assessment was defined as days from onset of HL to treatment start or, if no treatment was given, days to diagnostic audiogram

**Fig. S4 Sensitivity analyses of idiopathic SSNHL-cases for recovery^a^**

**A.** Multivariable linear regression for absolute recovery in dB

**B.** Multivariable logistic regression for full recovery

Abbreviations: CI: Confidence interval; HL: hearing loss; h: hours; dB: decibels; OR: odds ratio

A total of 236 patients with idiopathic SSNHL were included in the analyses, excluding 17 cases with an identified etiology post-diagnosis. ORs are displayed as logarithmic data for the purpose of data visualization

Age categories: Young adults: 18-39 years; Middle-aged adults: 40-59 years; Older adults: 60-69 years; Elderly: ≥70 years

Degree of HL: No/mild HL: ≤40 dB pure tone average of the frequencies 500, 1000, 2000, and 4000 Hz (PTA4); Moderate HL: 41-60 dB PTA4; Severe HL: 61-80 dB PTA4; Profound HL: >80 dB

^a^ Hearing recovery in A is defined as the absolute improvement in individual PTA (PTA of affected frequencies). In B, full recovery is defined as ≤10 decibels iPTA compared to the contralateral ear

^b^ B indicates the change in the outcome variable per 1 unit of change in the covariate. For categorical variables, B represents the effects of moving from the reference category

^c^ *P*-value <0.05 is considered statistically significant

^d^ Frequency range-specific HL: ≥30dB hearing loss in solely 3 or 4 consecutive frequencies

^e^ No treatment as reference

^f^ Disease duration until assessment was defined as days from onset of HL to treatment start or, if no treatment was given, days to diagnostic audiogram

| **Table S1** Clinical characteristics of patients | | | | | | |
| --- | --- | --- | --- | --- | --- | --- |
|  | All patients | | Corticosteroid treatment | | No treatment | |
| **Characteristic** | **Mean (SD)** | **No. (%)** | **Mean (SD)** | **No. (%)** | **Mean (SD)** | **No. (%)** |
| Age (years) | 57 (±17) | 253 | 56 (±17) | 207 | 63 (±17) | 46 |
| Young adults (18–39) |  | 51 (20) |  | 45 (22) |  | 6 (13) |
| Middle aged adults (40–59) |  | 66 (26) |  | 58 (28) |  | 8 (17) |
| Older adults (60–69) |  | 67 (27) |  | 55 (27) |  | 12 (26) |
| Elderly (≥70) |  | 69 (27) |  | 49 (24) |  | 20 (44) |
| Sex |  |  |  |  |  |  |
| Female |  | 115 (46) |  | 90 (44) |  | 25 (54) |
| Male |  | 138 (55) |  | 117 (57) |  | 21 (46) |
| Affected ear |  |  |  |  |  |  |
| Right |  | 109 (43) |  | 87 (42) |  | 22 (48) |
| Left |  | 144 (57) |  | 120 (58) |  | 24 (52) |
| Comorbidity |  |  |  |  |  |  |
| Hypertension |  | 91 (36) |  | 62 (30) |  | 29 (63) |
| Diabetes mellitus |  | 34 (13) |  | 19 (9) |  | 15 (32) |
| Associated symptoms |  |  |  |  |  |  |
| Subjective tinnitus |  | 127 (50) |  | 108 (52) |  | 19 (41) |
| Subjective dizziness |  | 57 (23) |  | 50 (24) |  | 7 (15) |
| Initial iPTA (dB) | 71 (±16) |  | 71 (±16) |  | 72 (±14) |  |
| Initial PTA4 (dB) | 68 (±23) |  | 68 (±24) |  | 66 (±23) |  |
| Degree of HL (PTA4, dB) |  |  |  |  |  |  |
| No/Mild HL (≤40) |  | 38 (15) |  | 32 (16) |  | 6 (13) |
| Moderate HL (41–60) |  | 51 (20) |  | 39 (19) |  | 12 (26) |
| Severe HL (61–80) |  | 80 (32) |  | 65 (31) |  | 15 (32) |
| Profound HL (≥81) |  | 84 (33) |  | 71 (34) |  | 13 (28) |
| Frequency range-specific HL^a^ |  | 51 (20) |  | 37 (18) |  | 14 (30) |
| Days to treatment or assessment | 6 (±7) |  | 5 (±6) |  | 10 (±8) |  |
| Full hearing recovery^b^ (iPTA) |  |  |  | 47 (23) |  | 11 (24) |
| Full hearing recovery (PTA4) |  |  |  | 61 (30) |  | 14 (30) |
| Abbreviations: SD: Standard deviation; No: number of patients; HL: hearing loss; PTA4: 4 frequencies pure-tone average at 500, 1000, 2000 and 4000 hertz hearing thresholds; iPTA: individual pure-tone average, average of affected frequencies; dB: decibels  All the % is of column total  ^a^ Frequency range-specific hearing loss is ≥30dB hearing loss in solely 3 or 4 consecutive frequencies  ^b^ Full hearing recovery is defined as ≤10 decibels PTA compared to the contralateral ear. | | | | | | |

**Fig. S5 One-way ANOVA for mean hearing recovery per affected frequency**

Descriptives represent number of affected frequencies (% of total patients)

* = P-value <0.05

** = P-value <0.01

**Fig. S6 Multiple unpaired t-tests for comparison of iPTA and PTA4 recovery in low- ^a^ and high-frequency ^b^ HL**


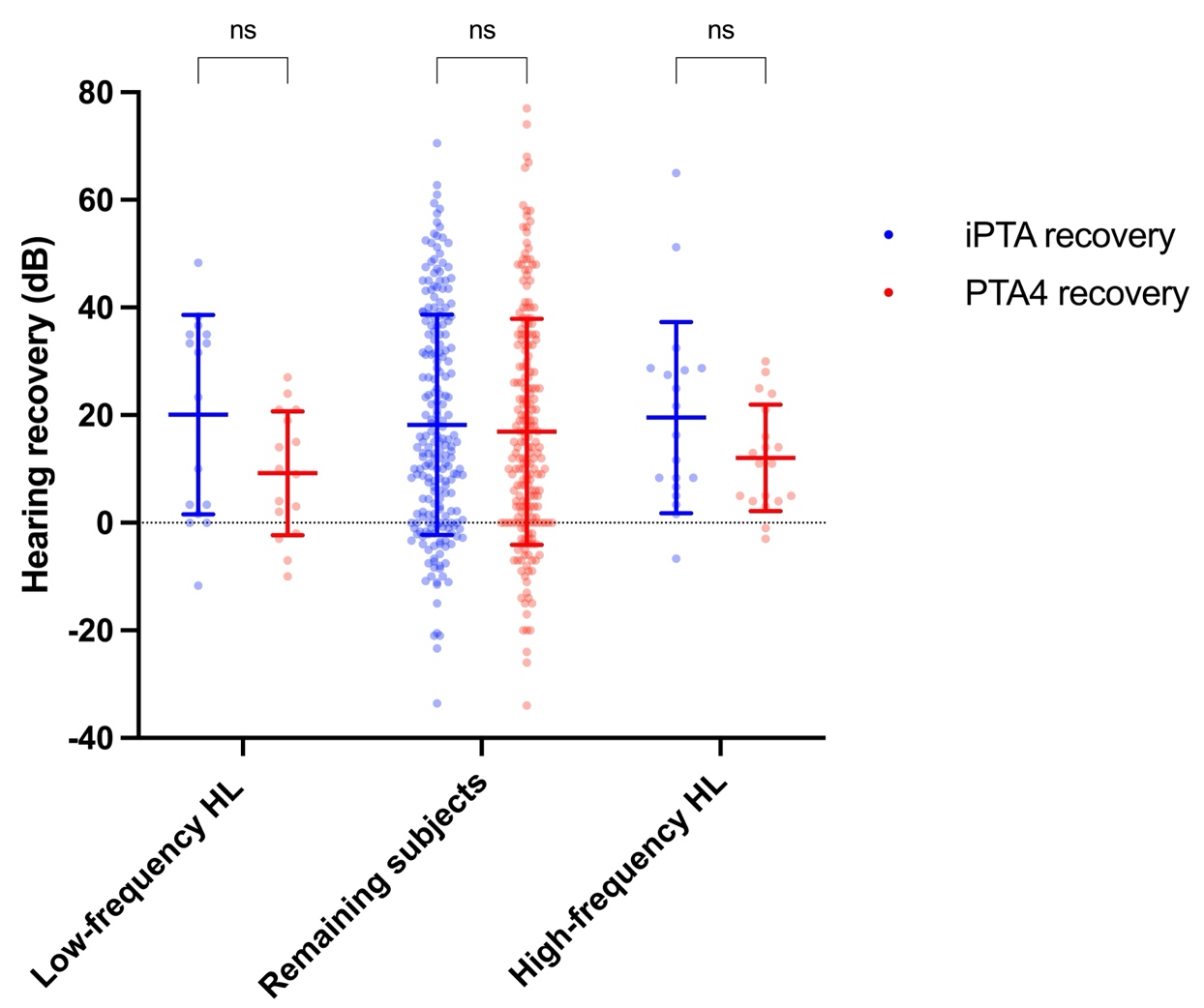


Abbreviations: iPTA; individual pure-tone average of solely affected frequencies; PTA4: pure-tone average of four frequencies, 500, 1000, 2000, and 4000 hertz; dB: decibels; HL: hearing loss

^a^ Low-frequency HL was defined as ≥30 dB HL at frequencies 125, 250, and 500 Hz

^b^ High-frequency HL was defined as ≥30 dB HL at frequencies ≥3000, 4000, 6000, and 8000 Hz
